# LoCKAmp: lab-on-PCB technology for <3 minute virus genetic detection†

**DOI:** 10.1039/d3lc00441d

**Published:** 2023-09-07

**Authors:** Sotirios Papamatthaiou, James Boxall-Clasby, Edward J. A. Douglas, Pawel Jajesniak, Hadrien Peyret, June Mercer-Chalmers, Varun K. S. Kumar, George P. Lomonossoff, Julien Reboud, Maisem Laabei, Jonathan M. Cooper, Barbara Kasprzyk-Hordern, Despina Moschou

**Affiliations:** a Department of Electronic and Electrical Engineering, University of Bath Bath BA2 7AY UK sp2216@bath.ac.uk; b Department of Chemistry, University of Bath Bath BA2 7AY UK; c Department of Life Sciences, University of Bath Bath BA2 7AY UK; d Division of Biomedical Engineering, James Watt School of Engineering, University of Glasgow Glasgow UK; e Department of Biochemistry and Metabolism, John Innes Centre, Norwich Research Park Colney NR4 7UH UK

## Abstract

The recent COVID-19 outbreak highlighted the need for lab-on-chip diagnostic technology fit for real-life deployment in the field. Existing bottlenecks in multistep analytical microsystem integration and upscalable, standardized fabrication techniques delayed the large-scale deployment of lab-on-chip solutions during the outbreak, throughout a global diagnostic test shortage. This study presents a technology that has the potential to address these issues by redeploying and repurposing the ubiquitous printed circuit board (PCB) technology and manufacturing infrastructure. We demonstrate the first commercially manufactured, miniaturised lab-on-PCB device for loop-mediated isothermal amplification (LAMP) genetic detection of SARS-CoV-2. The system incorporates a mass-manufactured, continuous-flow PCB chip with ultra-low cost fluorescent detection circuitry, rendering it the only continuous-flow μLAMP platform with off-the-shelf optical detection components. Ultrafast, SARS-CoV-2 RNA amplification in wastewater samples was demonstrated within 2 min analysis, at concentrations as low as 17 gc μL^−1^. We further demonstrate our device operation by detecting SARS-CoV-2 in 20 human nasopharyngeal swab samples, without the need for any RNA extraction or purification. This renders the presented miniaturised nucleic-acid amplification-based diagnostic test the fastest reported SARS-CoV-2 genetic detection platform, in a practical implementation suitable for deployment in the field. This technology can be readily extended to the detection of alternative pathogens or genetic targets for a very broad range of applications and matrices. LoCKAmp lab-on-PCB chips are currently mass-manufactured in a commercial, ISO-compliant PCB factory, at a small-scale production cost of £2.50 per chip. Thus, with this work, we demonstrate a high technology-readiness-level lab-on-chip-based genetic detection system, successfully benchmarked against standard analytical techniques both for wastewater and nasopharyngeal swab SARS-CoV-2 detection.

## Abstract 2

LoCKAmp: integrated LAMP genetic amplification device demonstrating reliable detection and ultrafast sample-to-answer time.

## Graphical abstract

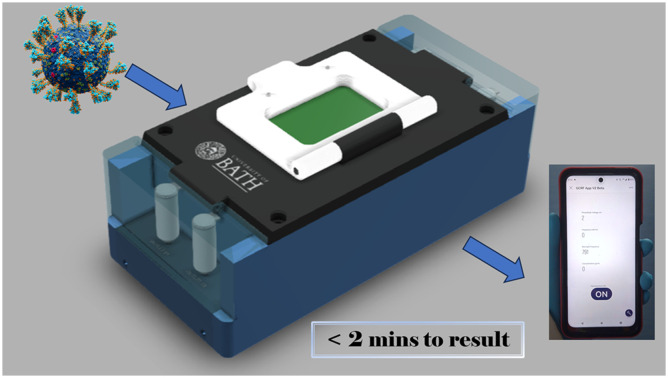

## Introduction

The need for more accurate monitoring of the spread of infectious diseases in the community was emphasized by the recent COVID-19 pandemic. The authorities lacked SARS-CoV-2 spread data of adequate granularity to effectively control the disease, due to limitations in existing testing capacity.^1^ The lack of diagnostic tests suitable for point-of-care (PoC) applications during the initial stage of the pandemic, along with the sensitivity limitations of the only test which has been used outside laboratory settings (*i.e.* the lateral-flow tests), led to ineffective monitoring of the virus transmission.^2^ The gap between the rapid but low-sensitivity and non-quantitative lateral-flow tests and the sensitive but expensive and time-consuming nucleic acid amplification tests (NAAT) has yet to be addressed, despite the huge resource influx in the area. Lab-based NAATs are predominantly polymerase chain reaction (PCR)-based assays, which is the gold standard, but requires complex and time-consuming sample processing and bulky thermocyclers with precise temperature control. Access to handheld and affordable NAAT would render the healthcare response prompt and precise in both developed and, crucially, low-resource settings with limitations in centralized analytical capacity. Miniaturized NAAT have shown potential with respect to sensitive, reliable and crucially rapid amplification time^2,3^ but still, lab-on-chip (LOC) technology has not managed to take these examples outside the lab into end-user hands.^4^ A key cause for the slow progress towards the real-life deployment of devices capable of hosting complex laboratory operations is the challenge in seamlessly integrating all the necessary steps, beginning with sample collection/pre-treatment and reagent manipulation up to the biosensing and the electronic control/communication. This seamless integration is key for device reliability (no inter-module connections) and cost-effective scalability through a standardized manufacturing technology.

Printed circuit boards (PCBs) may provide a realistic solution to these LoC integration and scalability challenges. Biosensing-related components integrated into PCBs, although first presented in 1996,^5^ have recently attracted increased attention from the research community,^4,6^ enough to expand the LoC field into the lab-on-PCB area.^7–9^ This is due to the recent focus on the integration strategies of individual LoC components into self-contained platforms, and the associated micro-fabrication technologies to achieve this lab-on-PCB technology being supported by the established expertise within the PCB industry, with regards to the industrial manufacturing processes of electronics.^10^ Additionally, there is extensive experience within the industry of micro-fabrication capabilities for fluidics implementation. These growing knowledge-bases within the industry have resulted in the system-level integration of microfluidic devices at a minimal cost. More specifically, lab-on-PCB devices range from flexible, wearable three-electrode electrochemical sensors for glucose sensing^9,11^ and DNA sensing^8^ to Bio-FETs^7^ and surface acoustic wave (SAW)-based acoustofluidics.^12^ Undeniably, miniaturised NAATs have been explored for PCB implementation with increasing numbers of prototypes reported to be matching standard, non-PCB devices or traditional benchtop methods.^13–15^ However, the vast majority of the miniaturised NAATs, regardless of whether a PCB platform is implemented, focus merely on the amplification reaction or/and the sample preparation (*i.e.* mixing, thermal lysis, extraction–purification). Generally, gel electrophoresis is used for the evaluation of the amplification product without any provision for real-time detection on a single platform. A holistic, sample-in-answer-out approach, in which the sample handling and genetic material amplification is integrated with a detection module in a practical system capable of deployment in the field, has yet to be demonstrated.^4,6,16–18^

In this paper, we report the development of the first such system (LoCKAmp), with an industrially manufactured lab-on-PCB device at its core, for ultrafast detection of severe acute respiratory syndrome coronavirus (SARS-CoV-2). The latter is a significant step forward from the previously published works on miniaturised NAATs on PCB,^14,15^ which may have used mass-manufacture compatible techniques but were made in-house. The technology can easily be extended to the detection of alternative pathogens or genetic targets for a very broad range of applications.

LoCKAmp is implementing reverse transcription LAMP (RT-LAMP) of SARS-CoV-2 with cell lysis and crucially, real-time fluorescence detection. All test elements are integrated in a compact and crude sample compatible system, with the test results demonstrated on a smartphone app. LAMP has become a preferable alternative to PCR, as it is more sensitive than conventional PCR, faster and more specific than quantitative PCR (qPCR), employing 4–6 primers to recognize multiple regions in the target sequence.^19,20^ Critically, it requires a single, stable temperature level, as opposed to three for PCR, eliminating the need for thermal cycling, thus reducing power consumption, and requires minimal nucleic acid purification,^21^ even in industrially manufactured microfluidic channels such as ours. Most published work on PoC LAMP detection of SARS-CoV-2 use chambers or tubes for colorimetric fluorescent detection,^22–24^ while our LAMP assay is performed under continuous flow in the microfluidic PCB chip, delivering faster time-to-result while reducing false positives *via* the increased sample flow-rates.^25^ The fluorescence detection integrated into LoCKamp employs off-the-shelf electronic components, demonstrating high sensitivity and quantitative results for 6 μL sample volume under continuous sample flow through a transparent tubing directly connected at the LAMP lab-on-PCB outlet. This is a significant advantage compared to the colorimetric detection in static compartment configurations,^22,23^ which may also produce quantitative results but show much lower sensitivity and much higher tendency for false positivity than our proposed approach.^26–30^

In this paper we demonstrate LoCKAmp's SARS-CoV-2 detection analytical performance in two very different, but equally critical, real-life applications: clinical nasopharyngeal samples and pre-processed wastewater samples. The aim is to show versatility of this technology in testing the same target in two contrasting samples: clinical (individual testing) and wastewater (community testing) to enable comprehensive public health surveillance. Wastewater-based epidemiology (WBE), utilising the concept of wastewater (capturing whole communities) as a fingerprint of community's health, has been adopted to track the spread of COVID-19 and other diseases faster than individual testing (even prior to active community cases with symptoms) and with less expense and intrusion.^31^ Expensive and centralised PCR analysis is the standard analytical method for WBE, which hampers it from unlocking its true potential as an early-warning testing-tool. Our device aims to progress the wastewater and clinical analysis field by fulfilling the need for a low-cost, rapid, sensitive and ultra-portable system for on-site SARS-CoV-2 detection. RNA extracted from pre-processed wastewater samples, using a PEG (polyethylene glycol) precipitation method, and thermally lysed patient nasopharyngeal swabs are both tested in this paper. These preparation methods were utilised due to our research lab biosafety level limitations in terms of handling live viruses. Nonetheless, full sample process on-chip is an application of great interest to further advance the wastewater lab-on-chip analysis field and an essential feature for diagnosis in clinical settings. Therefore, seamlessly integrated SARS-CoV-2 thermal lysis on-chip was also successfully implemented and presented in this paper, using virus-like particles (VLPs) encapsulating specifically designed portions of the SARS-CoV-2 genome as surrogates.

## Results and discussion

### Experimental setup – LoCKAmp system overview

**Fig. 1:**
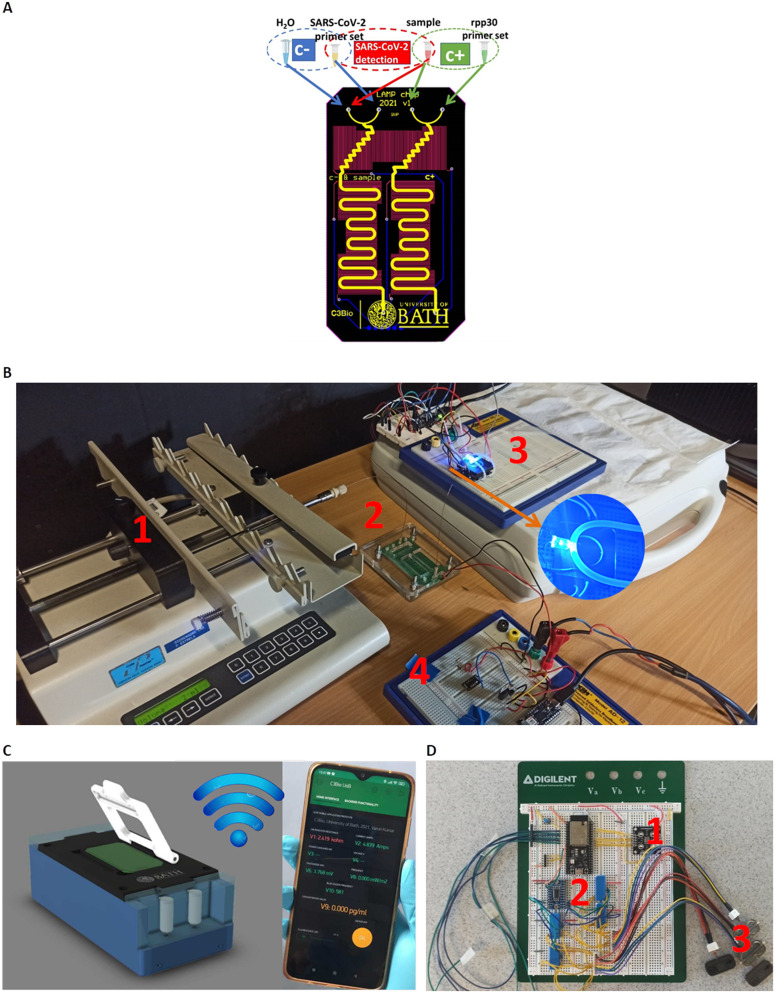
A) Process schematic of the 3-in-1 SARS-CoV-2 detection platform; input fluidic paths for the negative control (c−) (blue circle), the positive control (c+) (green circle) and the SARS-CoV-2 detection (red circle). The micromixer, the amplification channels and the outlet vias are displayed. Embedded, copper microheaters are displayed in red colour. B) The experimental setup of the ultrafast, real-time quantitative RT-LAMP platform: 1) syringe pump, 2) RT-LAMP on PCB, 3) fluorescence detection and 4) embedded microheaters controller. C) The CAD design of the portable instrumentation which includes all the system parts of the experimental laboratory setup in their compact version. Both the experimental setup and the portable version wirelessly transmit the test results *via* Wi-Fi to our custom mobile application. D) The prototype breadboard module with the control circuitry of the real-time quantitative RT-LAMP: 1) the optical sensor, 2) the control circuit for the heaters, the micro-pumps and the optical setup and, finally, the 3) Bartels mp6 micro-pumps.

Fig. 1A) shows the core component of the LoCKAmp system; the microfluidic PCB chip, capable of performing virus lysis and gene amplification under continuous flow. More specifically, it demonstrates the design of the full lab-on-PCB with the microfluidics and electronics. The micro-channel (in yellow) has a total length of 18.5 cm and has been designed to host the macro-to-micro interface, the virus thermal lysis component (micromixer) and the meandering channel for the RNA amplification. The system is designed for continuous flow operation and the channel height and width are 80 μm and 1 mm respectively, resulting in a total channel volume of 14.8 μL.

The embedded microfluidic channel is a critical feature for the efficient manufacturing of lab-on-PCB diagnostic devices, as the channel is designed and manufactured as a PCB layer and not separately. Resistive copper microheaters (red colour, 17 μm thickness) are embedded for sample heating^32^ while gold pads (blue colour) connect through spring-loaded pins with the low-power consumption (0.72 W at 5 V), battery operated, closed-loop control circuitry. The microheaters layer is placed behind the channel layer; they also meander, to provide the largest possible electrical resistance in the space available, thus ensuring sensitive temperature control.

The commercially manufactured PCBs were cleaned prior to use by means of a simple flush with nuclease-free water, following which no further pre-treatment was necessary. A negative control assay (blue group) is designed to run in parallel with the positive (green group) in their respective channels, followed by the sample analysis. The system is designed to accommodate four reagents in total: SARS-CoV-2 LAMP mix set and sample (or H_2_O when running negative control) and the rpp30 gene LAMP mix set and sample for the positive control.

Fig. 1B) shows the experimental setup for the LAMP platform. All reagents were injected into the PCB channel *via* a laboratory syringe pump apparatus at 2 μL min^−1^ (unless otherwise stated), using glass syringes to avoid flow-rate inaccuracies.

An acrylic chip holder was laser-machined and provided secure microfluidic interface to the fluidic PCB, while allowing space for connecting the chip with the heaters' control, Arduino circuitry. The PCB chip fluidic outlet was interfaced with a transparent tubing, mechanically fixed above the fluorescent detection module for the subsequent optical detection of the LAMP product, before being collected in a waste tube. Blue LED light provided optimal excitation light for the fluorescent dye employed (GelGreen®). A commercial light-to-frequency converter, controlled by a custom-made Arduino board, was used to detect the emission of the fluorescent dye (around 535 nm). An ultra-low cost, commercial filter was attached on top of the light-to-frequency converter, to effectively block the excitation LED but allow selectively the fluorescent dye emission wavelength to pass through. The light intensity change that is triggered by the LAMP product is detected by the converter as a change in frequency and the result is wirelessly transmitted *via* the Arduino (Wi-Fi) interface to our custom-made smartphone app. Fig. S1† illustrates the optical setup configuration visualizing the above descripted components.

This benchtop version is transferable to a portable instrumentation (Fig. 1C)), which essentially includes all the system parts of the experimental laboratory setup in a more compact version, as described by the block diagram in Fig. S2:† the fluorescence detection module (1), the on-chip heating control module (2), the digital control and transmission module (2) and the reagent flow-control module (3). In this portable version, the microfluidic chip is inserted in the handheld instrumentation where the miniaturized pumps, control circuitry and optical-detection circuitry reside to achieve a fully autonomous device. Straightforward, single-click interface between the PCB chip and the portable instrumentation has been achieved (patent pending). The full operation of the system (pumping, chip heating, digital control and optical detection) is controlled and monitored *via* an in-house developed smartphone application, where the test result is wirelessly transmitted to the smartphone. Fig. 1D) shows the prototype breadboard module with the control circuitry of the above-described operations and a first prototype, fully autonomous instrumentation, is shown in Fig. S3† with the μfluidic PCB in place.

### LAMP amplification assay optimization

**Fig. 2:**
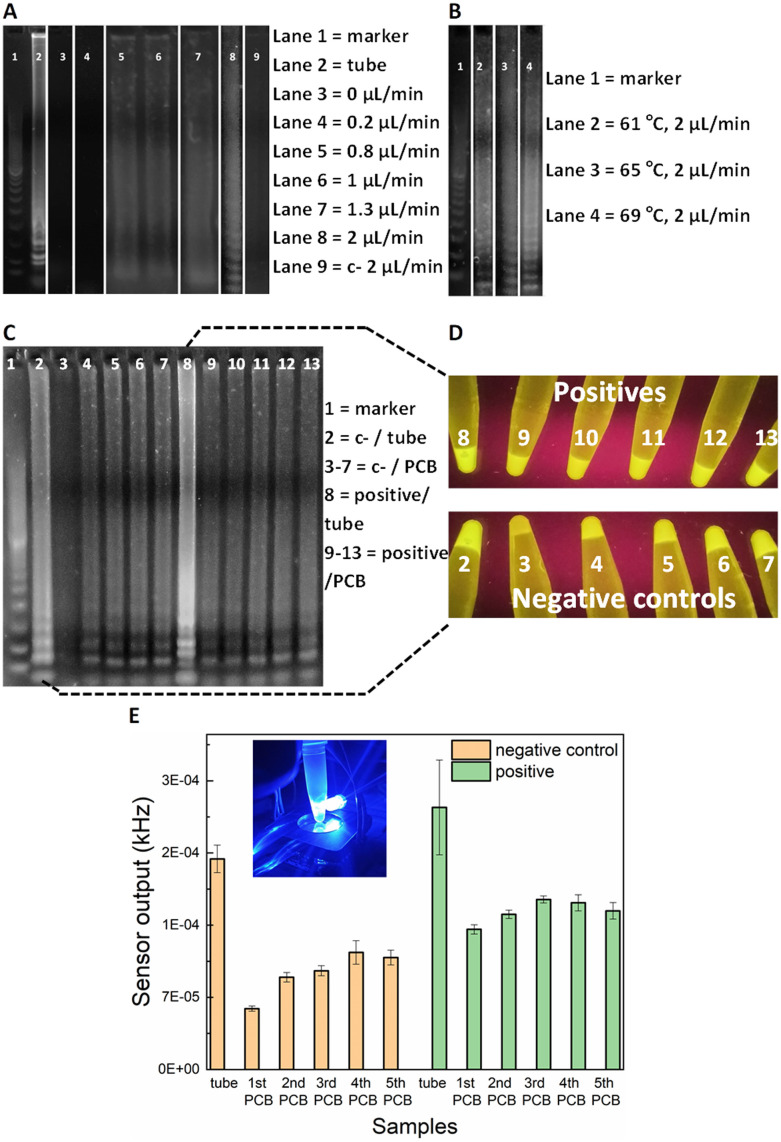
Agarose gel electrophoresis after performing LAMP on PCB A) at 65 °C using various flow rates (lane 2 shows product derived from the tube heated using dry-block incubator) and B) using 2 μL min^−1^ at various temperatures. DNA marker 100 bp is shown in Lane 1. Synthetic SARS-CoV-2 DNA was used as template (2 × 10^4^ gc μL^−1^). C) Lanes 2 and 8 show negative control and positive heated in tube (using dry-block incubator) and lanes 3–7 and 9–13 show negative controls and positives derived from the PCB, respectively. Two PCB channels were used for lanes 3–7 and 9–13, one for the negative controls and one for the positives, to evaluate the reusability of the PCB channel. Synthetic SARS-CoV-2 DNA was used as template (2 × 10^4^ gc μL^−1^). D) Photographic images of tubes under UV light which contain the remaining samples after the gel loading of Fig. 2C and E) optical sensor output for the same samples that are shown in Fig. 2D (bars represent standard deviations of three measurements for each tube).

For the on-chip validation of the miniaturised LAMP-on-PCB assay and definition of the optimal conditions, the on-chip reaction products were collected at the outlet and were initially assessed by gel electrophoresis to verify efficient amplification. ImageJ 1.52a software was used for semi-quantitative band intensity extraction. Synthetic SARS-CoV-2 DNA was used as template (2 × 10^4^ gene copies per μL). To optimise the protocol and obtain fast and efficient amplification, several reactions were run at various flow-rates in separate PCB fluidic channels at the Optigene-recommended 65 °C temperature (see Methods). In Fig. 2A), the agarose gel image displays the LAMP product retrieved for no flow (0 μL min^−1^), 0.2, 0.8, 1, 1.3 and 2 μL min^−1^ continuous flow rate for the on-chip LAMP and its comparison with the product derived from the off-chip (tube) amplification using a dry-block heater.

DNA amplification was successfully achieved in our chips for flow rates faster than, and including 0.8 μL min^−1^, whereas the static reaction and the 0.2 μL min^−1^ did not show any signal. The characteristic ladder pattern for LAMP product was observed.^20^ A negative control with the template replaced by H_2_O was passed, to assure our results were not a product of contamination. As the gel depicts, faster flow rates yield higher LAMP product. Indeed, the 2 μL min^−1^ band is 30% more intense than the 1.3 μL min^−1^ band and 50% than both the 1 and 0.8 μL min^−1^. The conventional, off-chip LAMP resulted in 22% more intense gel band than the 2 μL min^−1^, nevertheless it required 30 min of heating while the latter flow-rate lead to approximately 7 min amplification time to utilize 15 μL of product. This is in agreement with earlier studies on flow-through PCR, which demonstrated that the breakdown of mass transfer at small scales enables higher kinetics.^33^ Based on the flow-rate results, it was decided to perform the on-chip amplification at 2 μL min^−1^ for different temperature levels. The optimal temperature was subsequently defined to be 69 °C (Fig. 2B). More specifically, the band for 69 °C showed 23% more signal intensity than for 65 °C and 61 °C.

### Fluorescence sensor set-up

Having optimised the LAMP-on-PCB assay by defining the reaction temperature and the flow-rate of the continuous flow, the fluorescent detection setup was introduced. Here, the aim being to integrate the gene amplification with the detection module under continuous plug flow. In this part of the study, the LAMP product is still collected in Eppendorf tubes (10 μL) but instead of only being loaded on the gel, the samples were assessed *via* our fluorescence detection setup, by adding 10 μL of 3× GelGreen® fluorescent dye. Fig. 2C) highlights the sensitivity advantage of the light-to-frequency sensor compared to the gel electrophoresis and naked-eye detection. We initially performed five successive LAMP samples in the same PCB channel and compared the response obtained *via* each detection method. One PCB channel was dedicated to running negative control samples (lanes 3–7) and a separate one to run SARS-CoV-2 positive samples (lanes 9–13) (Fig. 2C)). The channels were flushed with H_2_O between sequential LAMP runs; this proved to be insufficient to remove reagent residues from the channels, as clearly demonstrated by the gel images. Gel lanes 4–7 are clearly false positives, demonstrating that reusing the same channels for subsequent sample analysis results in false positives. Nonetheless, the pristine channel negative control run (lane 3) is plainly distinguished from the pristine channel positive sample run (lane 9), clearly indicating that there are no issues with false positive in using pristine lab-on-PCBs. Placing the tubes under UV light (Fig. 2D)) for naked-eye detection resulted in the same observation, exactly matching the gel image: the negative samples injected in a previously used channel produce false positives while the initial negative control is visibly dimmer.

When these gel electrophoresis results are compared with the LoCKAmp fluorescent sensor response, we observe an interesting result (Fig. 2E)). Our optical sensing setup is able to differentiate every negative control from its positive counterpart, contrary to the previous two detection methods, making obvious the superior sensitivity achieved by employing the proposed optical detection setup. Even though an increase of the sensor output frequency values is noticed for the repeated runs, compared to the initial LAMP run, here the signal for the positives is clearly larger than the respective signal for the negative controls. This indicates and quantifies a cumulative effect when re-using the same channels, which could potentially be reversed with an appropriate cleaning technique, if required.

Fig. 2C) also shows a false positive result for off-chip amplification in gel lane 2 (*i.e.* tube number 2 in D) and left bar in E)). This false positive could be attributed to non-specific amplification or random self-amplification of the primer genes, a common characteristic reported for LAMP assays.^34^ Generally, isothermal amplification methods are considered more prone to false-positives than PCR^35,36^ as many of them work at low-temperature (between 30 and 55 °C). In addition, the LAMP reaction is extremely sensitive, thus small amounts of contamination can result in false positives. Throughout this work, 16% false positivity rate (25 samples in total) was noted for in-tube LAMP reactions, while no false positive generation was observed in pristine lab-on-PCBs (>55 runs in total) for on-chip amplification. We speculate that this is attributable to the much shorter analysis time required for our proposed flow-through on-chip reaction, compared to the >20 min static, in-tube implementation.

### Real-time detection of SARS-CoV-2 employing the LoCKAmp

After the promising results from the fluorescence detection setup, the transition to real-time detection of the RT-LAMP product under continuous flow (2 μL min^−1^) was performed. The PCB channel output was directly fed to the optical detection module, *via* transparent silicone tubing which was attached on the optical filter (Fig. S1†); a plug of 4 μL was assessed to be sufficient for recording a stable sensor output. The RT-LAMP mix was pre-combined with the GelGreen® before the amplification, as described in Methods.

### Assessment with heat-inactivated SARS-CoV-2

**Fig. 3:**
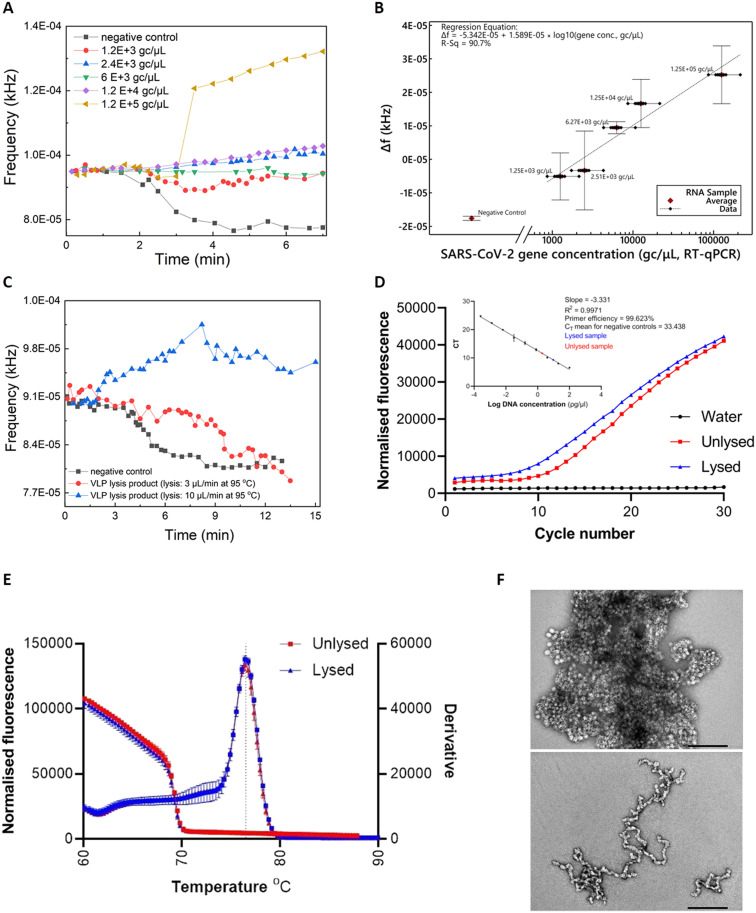
A) Real-time response for various SARS-CoV-2 RNA concentrations (inactivated SARS-CoV-2 viruses ATCC VR-1986HK). B) Mean values of Δ*f* (frequency_sample in tubing_ − frequency_empty tubing_) for various SARS-CoV-2 viral loads that correspond to clinical needs (bars represent standard deviations of 3 repeat experiments). C) Real-time response for RT-LAMP on PCB using the thermal lysis product of VLPs as template. The VLPs were lysed on PCB under 10 μL min^−1^ or 3 μL min^−1^ continuous flow. Unlysed VLPs were included as template for the negative control. D) RT-qPCR amplification. Inset shows the standard curve created by performing qPCR on nine 1 : 5 serially diluted DNA samples of synthetic SARS-CoV-2 gene. Data from RT-qPCR on lysed VLPs (red) and unlysed (blue) was added. DNA concentrations were subsequently interpolated from the standard curve. Dots represent the mean CT values and the error bars the standard deviation from two biological samples and technical duplicates. E) Melt curves comparison for lysed on PCB (10 μL min^−1^) and unlysed VLPs showing specific amplification (*T*_m_ = 76.42 °C). F) Negative stain transmission electron microscopy images for unlysed (top) and lysed (bottom) VLPs. Staining with 2% uranyl acetate, scale bars are 200 nm.

Commercially-acquired heat-inactivated SARS-CoV-2 RNA samples^37^ were first employed to assess the performance of our device. The real-time sensor response is illustrated in Fig. 3A), showcasing quantitative characteristics for RNA concentrations (ranging from 1.2 × 10^3^ to 1.2 × 10^5^ gc μL^−1^) covering the clinical range reported by Jones *et al.*^38^

The real-time fluorescent sensor response to the negative control sample is clearly distinguishable from the positive samples (Fig. 3A)). More specifically, frequency remains stable while the sample starts flowing out of the lab-on-PCB amplification module and into the outlet tubing, towards the middle point of the optical filter. This is the baseline signal, with the tubing over the sensor being empty or partially filled with sample. When the sample approaches the middle point of the sensor window, the sensor starts responding to the new state and its output signal will either decrease, if the sample is negative (LED light absorbed in non-fluorescing plug), or increase if it is positive and fluorescence was detected. In this figure, decrease of the sensor output is noted for the negative control, whereas a moderate or steep increase is demonstrated for three of the positive samples, with a shallow decrease for the 1200 and 6000 gc μL^−1^ positive samples. The response caused by all positive samples is clearly distinguishable from the negative control.

Fig. 3B) shows the calculated regression curve compared to RT-qPCR values. Δ*f* is the sensor output difference from the baseline signal (empty tubing). Bars represent standard deviations of 3 repeat experiments. A clear quantitative result is observed with 240 gc μL^−1^ limit of detection (considering the 3× SD of the negative control).

### SARS-CoV-2 virus-like-particle lysis, amplification and detection

Viral lysis is a crucial step towards realizing the vision of a sample-in-answer-out diagnostic device, expanding the integration capabilities of our system. Having successfully evaluated the amplification and detection part of LoCKAmp with real SARS-CoV-2 RNA, we proceeded with integrating a flow-through viral lysis module on our lab-on-PCB and evaluating its performance under continuous flow. Limited by our capability to handle live viruses in our laboratory, we proceeded with published SARS-CoV-2 virus-like particles (VLPs), incorporating the same RNA sequences we assessed in the work presented until now. Our VLPs served as a virus surrogate of known and controllable concentrations, having been previously verified to have similar behaviour to SARS-CoV-2.^39^

Two flow rates were investigated for thermal lysis of VLPs on chip to achieve different lysis time durations at 95 °C: 10 μL min^−1^ results in 1.5 min lysis time and 3 μL min^−1^ results in 5 min. These lysis times were reported by Ganguli *et al.*^24^ and Rabe *et al.*^40^ as successful thermal lysis strategies for SARS-CoV-2 at 95 °C. Then, the lysis products were used as RT-LAMP templates and run on PCB for the lysis verification. Fig. 3C) shows the real-time response of RT-LAMP on PCB under 2 μL min^−1^ continuous flow using the thermal lysis product of VLPs as template. Unlysed VLPs were included as a template for the negative control (black curve). A typical positive test response is seen for the VLP sample lysed at 10 μL min^−1^ (blue curve), whereas the 3 μL min^−1^ lysed sample (red curve) behaved similarly to the negative control. This shows that 1.5 min of heating at 95 °C successfully lysed the particles, whereas the increased lysis duration of 5 min possibly degraded the extracted RNA.^41^

RT-qPCR was also employed to further confirm the successful thermal lysis on chip, with Fig. 3D) and E) showing the amplification plots and melt curves. Indeed, the CT value for lysed (10 μL min^−1^) was 9.58 and for the unlysed 11.41, as Fig. 3D) inset shows. A 2 CT difference is less than the 9 CT reported by Peyret *et al.*^39^ who used the same types of VLPs (with a different packaged SARS-CoV-2 sequence), but they used chemical lysis and RNA purification instead of thermal lysis which surely helped to avoid having denatured VLP capsid protein floating in the sample potentially interfering with reverse transcription. The much higher VLP concentration used here (1000 ng μL^−1^*vs.* 0.5 ng μL^−1^) may have contributed to increase the possibility of lysing some particles during the first denaturation step of the PCR, thus amplifying the signal. Finally, TEM characterization on the VLPs was performed to observe the morphology differences between the lysed and the unlysed. Fig. 3F) reveals lysed on chip VLPs (bottom image) as clearly elongated particles with a tendency to form ring-like or open structures, compared to the unlysed (top image) which appear as spheres creating “large” clusters. The lysed particles are fused together, contrary to the clear separative character of the non-lysed.^39^

### SARS-CoV-2 detection in wastewater and flow-rate effect

**Fig. 4:**
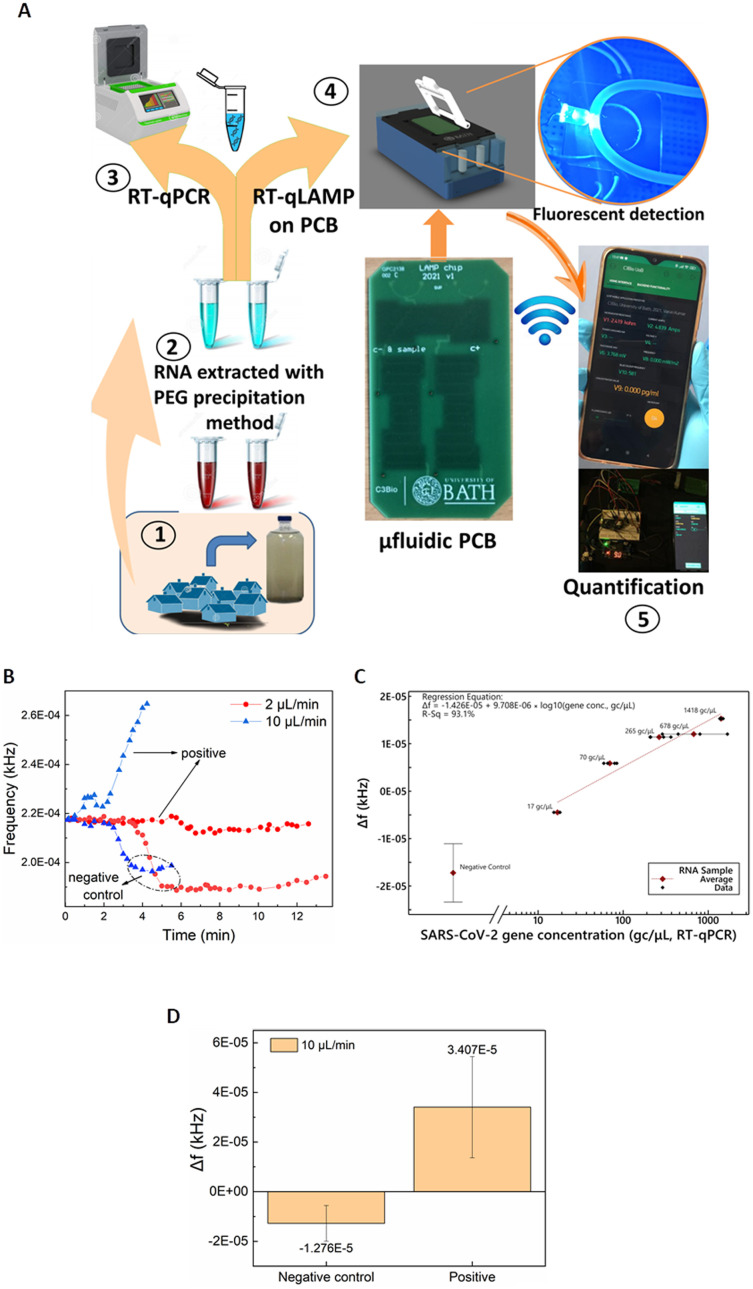
A) Overview of the LoCKAmp RT-LAMP device concept for real-time, quantitative SARS-CoV-2 detection in processed wastewater. Miniaturised RT-LAMP results were compared to standard RT-qPCR results. B) Real-time detection of SARS-CoV-2 in processed wastewater (17 gene copies per μL) and response comparison for 2 μL min^−1^ and 10 μL min^−1^ continuous flow rates on PCB. The negative control sample is the SARS-CoV-2 LAMP mix set with nuclease-free water used as the template. C) Linear regression for 2 μL min^−1^ continuous flow using SARS-CoV-2 RNA template in processed wastewater ranging from average (geometric mean) measured values (RT-qPCR) between 17 and 1418 gene copies per μL, plotted on a logarithmic scale; Δ*f* is the sensor output difference from the baseline signal (empty tubing). Error bars for the negative control represent standard deviation of 8 replicates. *R*^2^ = 93.1%. The linear regression excludes the negative control, as its inclusion causes the regression to be driven more by the value assigned to this ‘zero’ sample on a logarithmic axis than by the relative performances of the PCB and RT-qPCR techniques. D) Comparison of mean sensor output values for negative control and positive SARS-CoV-2 processed wastewater samples under 10 μL min^−1^ continuous flow; error bars represent standard deviation of 6 replicates for the negative control and 5 various RNA concentrations for the positive.

Having validated and benchmarked LoCKAmp's operation, we proceeded with testing its performance with real samples, pre-processed to inactivate SARS-CoV-2, due to laboratory biosafety restrictions. Fig. 4A) shows a schematic of the overview of the overall LoCKAmp device concept for real-time, quantitative SARS-CoV-2 detection in RNA extracted from wastewater. Wastewater samples were collected from a wastewater treatment plant located in South West England during the Covid outbreak (inset 1) and processed in the laboratory to extract RNA using PEG precipitation protocol (inset 2). RT-qPCR was then employed to quantify the SARS-CoV-2 concentration in the processed wastewater samples (inset 3). Subsequently, the samples were analysed using our LAMP assay in the microfluidic PCB chip (inset 4) and the test result was wirelessly transmitted to a smartphone (inset 5).

Fig. 4B) shows a representative example of how the real-time response of the light-to-frequency sensor differs for positive wastewater and negative control samples (nuclease-free water used as template) and for 2 μL min^−1^ (red colour) and 10 μL min^−1^ (blue colour) continuous flow rates. Here, decrease of the sensor output is noted for both the negative controls whereas a steep increase is demonstrated for the 10 μL min^−1^, with a shallow decrease for the 2 μL min^−1^ positive samples. Normally, a substantial increase is observed for any positive sample regardless of which of the two flow-rates is used but Fig. 4B) shows the response to the lowest tested concentration of RNA in wastewater: 17 gene copies per μL. It is obvious that the magnitude of the response for the faster flow for the positive sample is greater than the respective response for the slow flow rate. On the contrary, a smaller Δ*f* decrease for the faster flow rate compared to the slower is observed for the negative control samples. This pattern was noticed for all the tested samples regardless of the RNA concentration. The reaction output signal of our sensor is more pronounced for the 10 μL min^−1^ flow rate. The flow rate dependent response comparison (Fig. S4†) further confirms the findings presented above in Fig. 2 where the static reaction did not produce any signal.

Our hypothesis for this flow-rate effect on reaction time is that the prevailing mechanism is the decrease in the geometric dimensions of this diffusion-limited reaction in our lab-on-PCB (compared to the cm-scale dimensions for the in-tube reactions). This is in combination with non-specific adsorption of reaction reagents in the increased surface area, or the potential presence of amplification inhibitors. Further investigation to shed light into the underlying mechanisms resulting in this flow rate effect, is required.

The calculated calibration for our system compared to benchtop RT-qPCR values is shown in Fig. 2C). 2 μL min^−1^ continuous flow was used for various template concentrations ranging from 17 to 1418 gene copies per μL. Error bars for the negative control represent standard deviation of 8 replicates. The lack of exact same SARS-CoV-2 concentrations in real wastewater samples did not allow replicates for the positive samples. A distinct quantitative result is observed with 41 gene copies per μL limit of detection (considering the 3 × SD of the negative control). The response under 10 μL min^−1^ continuous flow produced distinct identification of SARS-CoV-2 positive samples in all cases. Fig. 4B) depicts an indicative curve for all positive samples), nonetheless the sensor output did not prove to be proportional to the laboratory anticipated RNA concentrations. Therefore, a cumulative comparison between all the various concentrations of positive samples (5 samples ranging from 17 to 1418 gc μL^−1^) and six replicates of negative controls is presented in Fig. 4D). Nonetheless, the positives are clearly distinguished from the negative controls, even at this fast flow rate, making the setup suitable for applications that demand faster sample-to-answer time and a yes/no rather than a quantitative test answer.

Taking into consideration the time needed between the moment the sensor starts reacting to the fluid passing over it until its signal reaches a plateau, approx. 4 μL are needed to confirm the negative test result. Considering this, the sample-in to answer-out response of the system is approx. 7 min when 2 μL min^−1^ flow-rate is used and 2–3 min for the faster flow-rate of 10 μL min^−1^. This indicates that the sample-to-answer time could be further reduced below 2 min, when an optimized experimental setup is implemented with respect to reducing the dead volumes between the chip and the sensor. To the best of our knowledge, this renders our chip the fastest reported so far, with respect to total analysis time.^14,15,24,42^

### SARS-CoV-2 detection from patient samples

The next and final step in this presented study was to evaluate our system's performance when using raw patient samples, benchmarking it against the current clinical standard for SARS-CoV-2 detection. The detailed clinical study workflow is presented in Fig. S5.† Nasopharyngeal patient samples were collected at Royal United Hospitals (RUH) Bath hospital and were subjected to RT-PCR locally. Ten (10) negative and ten (10) positive samples were collected by the research nurses, while a second identical swab sample for each patient was retrieved and transferred to our laboratory in 2 mL 1× PBS tubes. These were subjected to short thermal off-chip lysis at 95 °C for 5 min (ref. 40) and subsequently analyzed with LoCKAmp without any purification/RNA isolation protocol or any other type of sample preparation. Half of the thermally inactivated sample volumes were sent to the University of Glasgow for RT-qPCR analysis, blinded. The results are summarized in Table 1. Good agreement with the RUH measurements were shown with 70% positive percent agreement (PPA)^43^ and 100% negative percent agreement (NPA). Considering the intermediate thermal inactivation step, there was some variation between the RT-PCR of RUH and the RT-PCR of University of Glasgow (70% agreement for the positive samples and 100% for the negatives). When benchmarked against the RT-PCR analysis of the thermally inactivated samples at the University of Glasgow, LoCKAmp showed improved sensitivity (85.7%) and similar selectivity (92.3%) compared to the results before the virus deactivation.

**Table 1:** Clinical validation of RT-LAMP lab-on-PCB device for real-time COVID-19 detection using nasopharyngeal swabs compared to the reference standard RT-PCR method employed at RUH Bath and University of Glasgow. RT-PCR was initially performed at RUH Bath and a second swab was supplied for the lab-on-PCB RT-LAMP and RT-PCR at University of Glasgow. PPA positive percent agreement, NPA negative percent agreement^43^

RT-LAMP lab-on-PCB (LoCKAmp)		RT-PCR performed at RUH Bath		RT-PCR performed at University of Glasgow	
Positive	Negative	Positive	Negative	Positive	Negative
7	13	10	10	7	13
PPA%		70		85.7	
NPA%		100		92.3	

## Conclusions

LoCKAmp is a complete diagnostic system centred on the core-lab-on-PCB technology for rapid genetic amplification. Initially, the system was evaluated for the detection of SARS-CoV-2, proving its practicality and applicability in real-life deployment. This approach can easily be extended to the detection of alternative pathogens or genetic targets for a very broad range of applications. This system offers the capability to perform real-time, quantitative LAMP SARS-CoV-2 detection using an instrumentation setup enabling a small, portable platform. It seamlessly integrates the continuous-flow, miniaturised LAMP PCB chip with a simple, ultra-low cost optical detection module and a seamlessly integrated thermal lysis module. The core, disposable element of our platform is the PCB chip which is manufactured with fully upscalable techniques in a standarised PCB factory at a small scale production cost of £2.50 per chip. Successful SARS-CoV-2 detection and quantification in wastewater-derived RNA samples was demonstrated within 7 min with viral load information, or 2–3 min when a yes/no answer was required, at concentrations as low as 17 gc μL^−1^. To our knowledge, this makes our chip the fastest ever reported.^14,15,24,42^ Crucially, our LAMP on PCB exhibited superior reliability compared to the off-chip reaction, without any false positive results. The clinical study successfully exploited patient samples, further expanding the functionality of our system for individual patient testing. LoCKAmp did not require any time-consuming sample preparation steps when analyzing patient nasopharyngeal swabs, requiring only a rapid thermal lysis step to release SARS-CoV-2 RNA. Successful on-chip thermal lysis of SARS-CoV-2 RNA-containing virus-like-particles was reported within 1.5 minutes and confirmed by classical RT-qPCR. The lysis product was successfully amplified on PCB and detected by our optical setup under continuous flow.

LoCKAmp convincingly addresses some of the longest-standing challenges in lab-on-chip research and paves the way for the widespread technology adoption in real-life. It proves that rapid laboratory-level molecular diagnostics outside a controlled laboratory setting are truly achievable and practical, by far exceeding existing lateral flow tests in terms of time-to-result and performance,^44^ thus setting a new basis for point-of-care diagnostic technology.

## Materials and methods

### Microfluidic PCB

The custom-made lab-on-PCB was designed in Altium® (adapted from ref. 45) and was mass-manufactured in an established, standardized factory (Graphic PLC, UK) (£2.50 per chip). The board dimensions are 4.4 cm × 8 cm. It implements an embedded, double-channel layout (FR4 type 106, routed to create channel for fluids) for continuous flow operation incorporating the SARS-CoV-2 test with positive and negative controls.

### Setup for continuous flow RT-LAMP and real-time optical detection

The LAMP mix was flowed through the channel by a syringe pump (Cole-Parmer, USA) at 2 μL min^−1^ unless otherwise stated. GelGreen® Nucleic Acid Gel Stain was employed as fluorescent dye. The light-to-frequency convertor was the TCS230 and the optical filter was the Accuris SmartDoc Filter, attached on top of the TCS230. The test result is transmitted to the user's smartphone using a custom-made project in the Blynk IoT app. For independent temperature measurements on miniaturised LAMP during microheater operation, a thermal camera (FLIR TG165) was utilized. The miniaturised version of the system replaced the syringe pump with the mp6 micropumps by Bartels Microtechnik.

### LAMP sample preparation

Initial evaluation of our device for LAMP amplification was performed with a 2450-bp synthesised plasmid (pEX-A128) supplied by Eurofins Scientific. LAMP primers were designed^32^ by aligning genome sequences of SARS-CoV-2 in the ORF1ab (region spanning nucleotides 3031 to 3285 based on sequence with accession number MW922022.1), as recommended by the Chinese Centre for Disease Control and were purchased from Sigma-Aldrich. LAMP reactions were optimized in a final volume of 25 μL containing 15 μl of ISO-004 or ISO-004-RT Mastermix (Optigene, UK),^34^ 5 μl of template, 0.8 μM of inner primers (FIP/BIP), 0.4 μM of loop primers (LF/LB) and 0.16 μM of external primers (F3/B3). The primers set comprised the following sequences: F3: 5′-TCC AGA TGA GGA TGA AGA AG-3′, B3: 5′-CAA CAA TTG TTT GAA TAG TAG-3′, FIP: 5′-AGA GCA GCA GAA GTG GCA CGG TGA TTG TGA AGA AGA AGA GT-3′, BIP: 5′-TCA ACC TGA AGA AGA GCA AGA AGT CTG ATT GTC CTC ACT GCC-3′, FLOOP: 5′-CTC ATA TTG AGT TGA TGG CTC-3′, BLOOP: 5′-CAA ACT GTT GGT CAA CAA GAC-3′.

### Stain protocol for gel electrophoresis and fluorescent sensor detection

Agarose gel electrophoresis was used to evaluate and optimise the on-chip LAMP before transitioning to the real-time fluorescent detection setup. LAMP products were loaded on a 2% agarose gel stained with GelGreen® and visualized with an ultraviolet (UV) transilluminator (Accuris™ SmartDoc™). 5 μL of the collected flow-through LAMP product were mixed with 1 μL of UView™ 6× loading dye (Bio-Rad) and were loaded on the gel. EZ Load 100 bp PCR Molecular Ruler (Bio-Rad) (5 μL) was used as a reference DNA marker. Electrophoresis was performed using a Bio-Rad power supply system for 40 min at 110 V.

The real-time, fluorescent detection setup mandated the inclusion of the dye in the reaction mix, avoiding any post-amplification staining as the detection happened sequentially with the amplification under continuous flow in a closed system. More specifically, 25 μL of GelGreen® diluted in H_2_O (from 10 000× to 3×) was added to the reaction mix, thus doubling the total reaction volume to 50 μL.

### RT-PCR of wastewater samples

Influent wastewater samples were collected from various wastewater-treatment plants in the South-West UK, upstream of any treatment processes. Samples were collected using automated 24 hour composite sampling in a flow- or time-proportional manner, whereby sampling was carried out every 15–30 minutes for 24 hours, with the sample size correlated with flow-rate for flow-proportional samples. Samples were processed at the University of Bath using a polyethylene glycol-based viral concentration and precipitation protocol, whereupon purified viral RNA was prepared in nuclease-free water *via* a TRIzol™-based extraction protocol.^46^ Where relevant, viral RNA was also extracted from non-wastewater samples *via* this same TRIzol™-based protocol: *i.e.* samples of commercially acquired, thermally inactivated SARS-CoV-2 (ATCC VR-1986HK).^37^ SARS-CoV-2 nucleocapsid gene and envelope gene concentrations were quantified by duplicate analysis in multiplex as per assays used by the US Centres for Disease Control^47^ (N1 gene target) and Corman *et al.*^48^ (E-Sarbeco gene target), respectively. Analysis was carried out at the University of Bath by RT-qPCR on an AriaMX Real-Time PCR machine (Agilent) using FAM and HEX for SARS-CoV-2 N1 and E-Sarbeco gene-target detection respectively. SARS-CoV-2 concentration was calculated from the quantified N1 and E-Sarbeco gene target concentrations, using a weighted geometric mean calculation wherein the weights were inversely proportional to the analytically determined %CV of each gene target.

### RT-PCR of clinical samples

Twenty (20) thermally-inactivated and blinded nasopharyngeal patient samples were delivered to the University of Glasgow and analyzed by RT-qPCR using Luna SARS-CoV-2 RT-qPCR Multiplex Assay Kit (New England Biolabs), according to the manufacturer's recommendation. In brief, 4 μl of each sample was mixed with 5 μl Luna Probe One-Step RT-qPCR 4× mix, 2 μl SARS-CoV-2 primer/probe mix (N1/N2/RP) and 9 μl nuclease-free water, and underwent thermocycling using QuantStudio 5 Real-Time PCR System (Thermo Fisher Scientific). Each sample was tested in duplicate for the presence of two SARS-CoV-2-specific targets, N1 and N2, using distinct fluorescent probes/channels (HEX and FAM, respectively). As the N2 target did not amplify for any of the samples, only N1 target was used in in the evaluation of patient samples.

### VLPs preparation and thermal lysis investigation

The target SARS-CoV-2 RNA sequence was specifically encapsulated into VLPs of the plant-infecting cowpea mosaic virus, and these VLPs were extracted and purified exactly as described in Peyret *et al.*^39^ Complete sequence: UCC AGA UGA GGA UGA AGA AGA AGG UGA UUG UGA AGA AGA AGA GUU UGA GCC AUC AAC UCA AUA UGA GUA UGG UAC UGA AGA UGA UUA CCA AGG UAA ACC UUU GGA AUU UGG UGC CAC UUC UGC UGC UCU UCA ACC UGA AGA AGA GCA AGA AGA AGA UUG GUU AGA UGA UGA UAG UCA ACA AAC UGU UGG UCA ACA AGA CGG CAG UGA GGA CAA UCA GAC AAC UAC UAU UCA AAC AAU UGU UGA GGU UCA ACC UCA AUU AGA GAU GGA. After pelleting by ultracentrifugation, VLPs were treated with micrococcal nuclease to digest any non-packaged nucleic acid, then buffer-exchanged thoroughly into 10 mM sodium phosphate, pH 7.2, then supplemented with sodium azide to 3 mM. The BCA assay was used to quantify VLPs at 23.6 mg mL^−1^. On-chip thermal lysis of the VLPs was performed using 50 μL of VLP solution in H_2_O at a concentration of 1000 ng μL^−1^. More specifically, the VLP solution was continuously flowed in the microfluidic PCB channel while the microheaters generated stable 95 °C temperature level.

Negative stain transmission electron microscopy was employed to characterise the VLPs morphology before and after the thermal lysis on-chip. The thermal lysis product was evaluated by both RT-LAMP on-chip and off-chip RT-qPCR. The latter was performed as follows: reverse transcription of viral RNA to complementary DNA (cDNA) was achieved in a one-step reaction using SuperScript™ III Platinum™ (Invitrogen) according to the manufacturers protocol with slight modifications. Instead of using random hexamers, primers ORF1ab FW 5′-TCCAGATGAGGATGAAGAAG-3′ and ORF1ab RV 5′-AGCAGCAGAAGTGGCAC-3′ which were specific for the ORF1ab region of SARS-CoV-2 were employed. To establish primer efficiency, standard curves were generated over a range of nine 1 : 5 serially diluted concentrations (2.5 ng μl^−1^–0.0064 pg μl^−1^) using the above primer set against purified ORF1ab from the pEX-A128 plasmid. The RT-qPCR reaction was performed as follows: 2 μl of ORF1ab template DNA or SARS-CoV-2/VLP cDNA, 10 μl of 2× SYBR™ Green PCR Master Mix (applied biosystems), 1 μl of 10 μM forward primer, 1 μl of 10 μM μl reverse primer and 6 μl of UltraPure™ DNase/RNase free distilled water (Invitrogen) in a 20 μl total volume. The RT-qPCR was performed on an applied biosystems StepOnePlus™ real time system and the cycling conditions consisted of an initial denaturation step of 95 °C for 2 min, followed by 40 cycles of two step cycling: 95 °C for 15 s, 60 °C for 1 min. Cycle thresholds (CT) were determined for two biological replicates in duplicate. Viral cDNA concentration was calculated through standard curve interpolation.

## Author contributions

Sotirios Papamatthaiou: conceptualization, formal analysis, investigation, visualization, methodology, writing – original draft and editing. James Boxall-Clasby and Edward J. A. Douglas: investigation, formal analysis, visualization and writing – review & editing. Pawel Jajesniak: investigation, formal analysis and writing – review & editing. Hadrien Peyret: investigation and writing – review & editing. June Mercer-Chalmers: methodology, project administration and writing – review & editing. Varun K. S. Kumar: software and investigation. George P. Lomonossoff, Julien Reboud, Maisem Laabei, Jonathan M. Cooper and Barbara Kasprzyk-Hordern: resources, supervision and writing – review & editing. Despina Moschou: conceptualization, methodology, visualization, funding acquisition, project administration, resources, supervision and writing – review & editing.

## Conflicts of interest

There are no conflicts to declare.

## Supplementary Material

LC-023-D3LC00441D-s001
