# Supplementary material for: LoCKAmp: lab-on-PCB technology for <3 minute virus genetic detection
Source: Lab Chip. 2023 Sep 7;23(20):4400–12. doi: 10.1039/d3lc00441d (PMC10563828; doi:10.1039/d3lc00441d)
Supplement: LC-023-D3LC00441D-s001 [file LC-023-D3LC00441D-s001.pdf]

Electronic Supplementary Material (ESI) for Lab on a Chip.
This journal is © The Royal Society of Chemistry 2023

LoCKAmp: Lab-on-PCB technology for <3 minute virus genetic detection

Sotirios Papamatthaiou,*[a] James Boxall-Clasby,[b] Edward J.A. Douglas,[c] Pawel Jajesniak,[d] Hadrien Peyret,[e] June Mercer-Chalmers,[a] Varun K. S. Kumar,[a] George P. Lomonossoff,[e] Julien Reboud,[d] Maisem Laabei,[c] Jonathan M. Cooper,[d] Barbara Kasprzyk-Hordern[b] and Despina Moschou[a]

** corresponding author*

[a]*Department of Electronic and Electrical Engineering, University of Bath, Bath BA2 7AY, UK.*
[b]*Department of Chemistry, University of Bath, Bath BA2 7AY, UK.*
[c]*Department of Life Sciences, University of Bath, Bath BA2 7AY, UK.*
[d]*Division of Biomedical Engineering, James Watt School of Engineering, University of Glasgow, Glasgow, UK.*
[e]*Department of Biochemistry and Metabolism, John Innes Centre, Norwich Research Park, Colney, NR4 7UH, UK.*

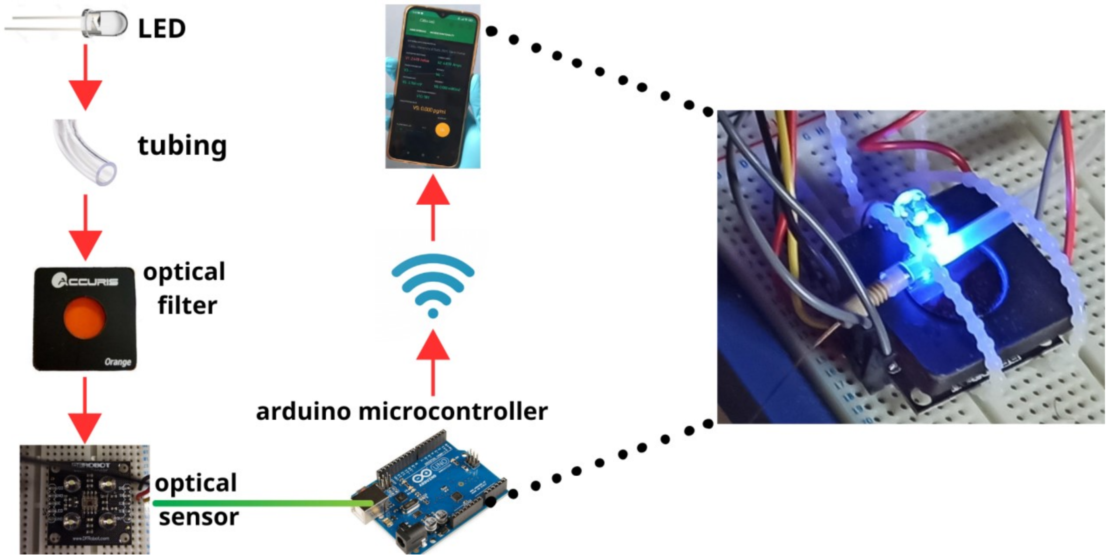

Supplementary figure 1: The working principle of the developed optical setup.

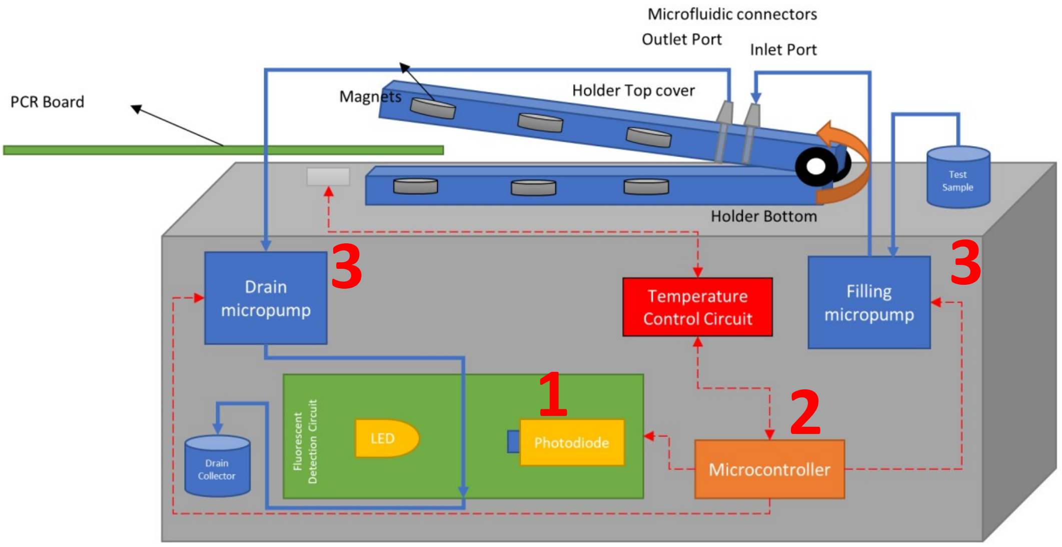

Supplementary figure 2: Block diagram of the portable LoCKAmp showing the 1) optical detection circuit/modules, 2) the control circuit for the heaters, the micropumps and the optical setup and finally 3) the micropumps.

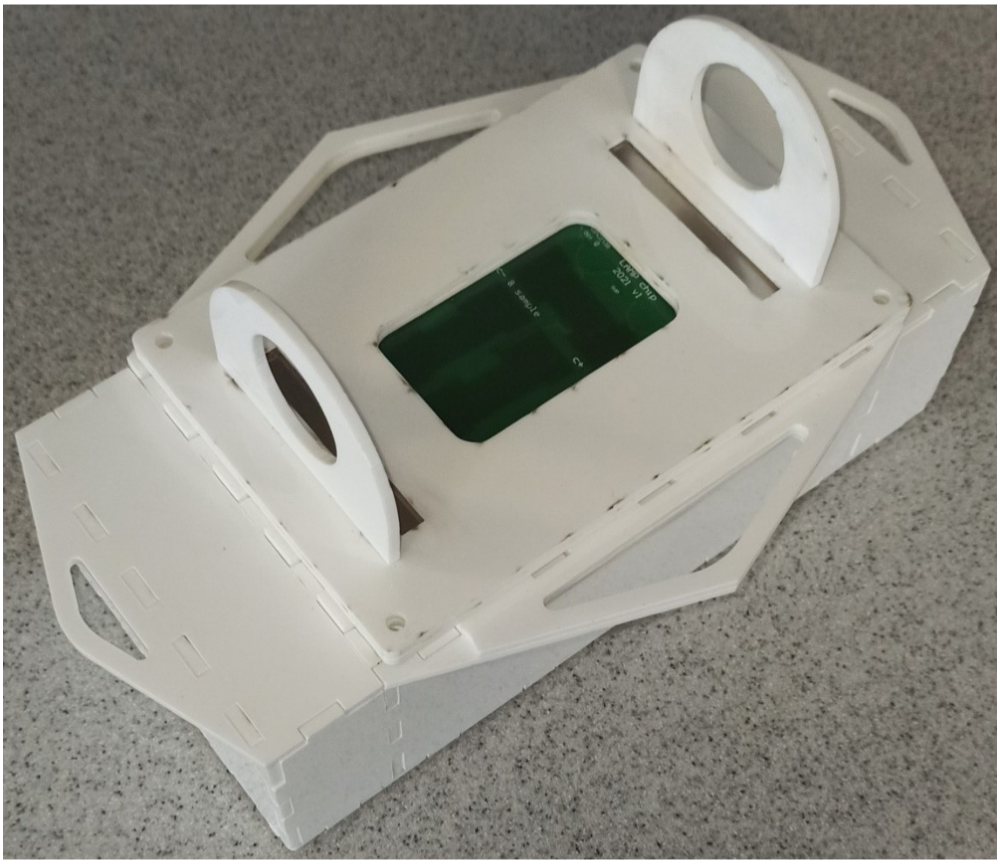

Supplementary figure 3: Photo of the prototype handheld instrumentation where the miniaturized pump-control and optical-detection circuitry reside. The micro-fluidic PCB is visible at the centre.

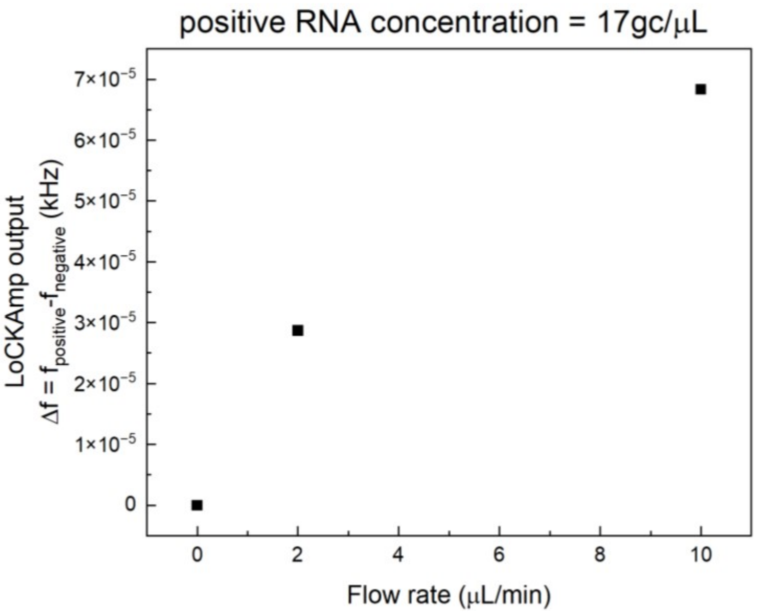

Supplementary figure 4: Dependency of the fluorescent sensor response on the flow rate of the μLAMP continuously flowed on the PCB.

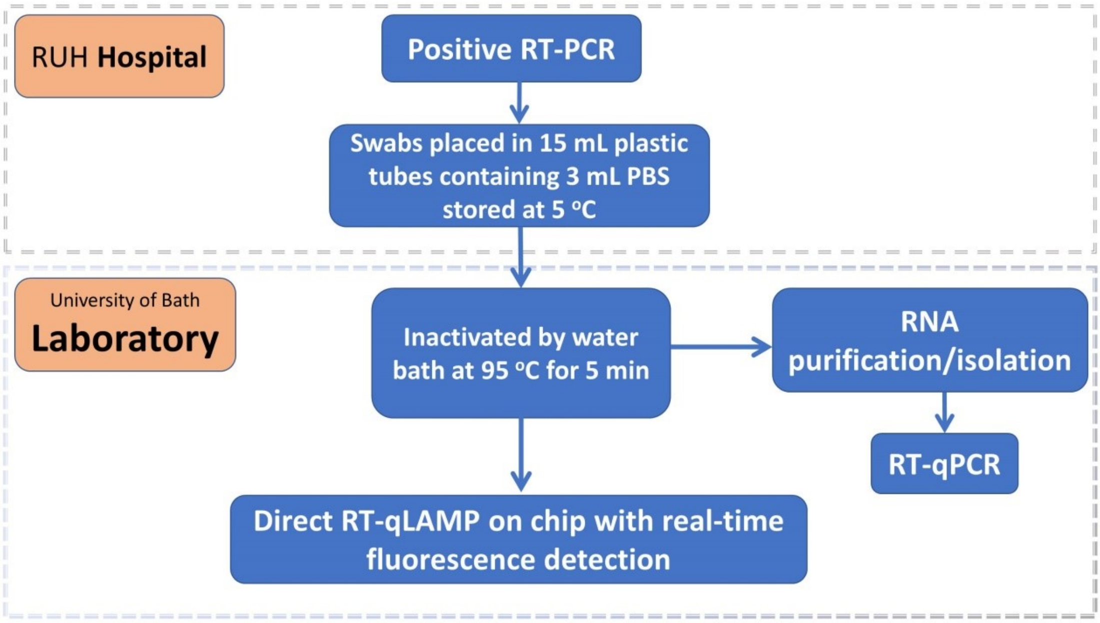

Supplementary figure 5: Clinical study work-flow. Nasopharyngeal samples taken at hospital and transferred to the laboratory were subjected to thermal lysis. Samples were submitted to both RT-qLAMP on chip and conventional RT-qPCR for correlation in the research laboratory.
